# Effects of thickened drinks on the disintegration of various oral tablets

**DOI:** 10.1016/j.heliyon.2020.e05800

**Published:** 2020-12-22

**Authors:** Taisuke Matsuo, Chinatsu Sato, Takashi Tomita, Yasuyuki Sadzuka

**Affiliations:** a Division of Advanced Pharmaceutics, Department of Clinical Pharmaceutical Sciences, School of Pharmacy, Iwate Medical University; 1-1-1 Idaidori, Yahaba-cho, Shiwa-gun, Iwate 028-3694, Japan; b Department of Pharmaceutical Sciences, Faculty of Pharmaceutical Sciences, Teikyo Heisei University; 4-21-2, Nakano, Nakano-ku, Tokyo 164-8530, Japan

**Keywords:** Disintegration time, Dysphagia, Food thickener, Tablet, Thickened drink, Xanthan gum

## Abstract

Food thickeners are widely used to aid the oral administration of medications to patients with dysphagia. Powder-type food thickeners are used to modulate the viscosity of therapeutic solutions depending on the swallowing capacity of patients. Food thickeners inhibit or delay the disintegration of some medications, resulting in reduced pharmaceutical effects of the medications and/or their excretion in the stool. A short immersion time (within 1 min) is important to overcome these problems. Although thickened drinks are commercially available, their use as vehicles for medications is not well understood. In this study, we evaluated the effects of thickened drinks on the disintegration time of therapeutic tablets. Furthermore, we compared the thickened drinks with powder-type xanthan gum-based food thickeners. Forty tablets were used, including naked tablets, film-coated tablets, orally disintegrating tablets, enteric-coated tablets, and sugar-coated tablets. For the disintegration test, the tablets were immersed in thickened drinks or food thickeners for 1 min. The changes in the disintegration time of the 40 tablets immersed in the thickened drinks were comparable with those in food thickeners. The disintegration time of several tablets was shorter or unchanged after immersion in the thickened drinks. The disintegration time of rapidly disintegrating tablets tended to increase when immersed in thickened drinks, but it was less than 2 min for the majority of the tablets. These results demonstrate that thickened drinks, similar to food thickeners, could help administer medications to patients. Overall, our study provides valuable information for pharmacists and clinicians to decide the most suitable way to deliver medications to patients with dysphagia.

## 1 Introduction

Elderly patients with dysphagia may experience problems in eating and drinking normally; furthermore, watery and low viscosity foods and drinks such as water, tea, and miso soup pose a potential risk of aspiration. Powder-type food thickening agents (food thickeners) are widely used to overcome these problems. Food thickeners prevent accidental aspiration by reducing swallowing speed [1]. Food thickeners are classified as mildly, moderately, and extremely thick [2], and individuals with dysphagia can prepare thickened drinks of appropriate concentrations based on their needs. Food thickeners can also be classified as starch-type, guar gum-type, and xanthan gum-type, depending on their formulation [3]. Xanthan-gum-based thickeners are frequently used for patients with dysphagia in nursing facilities, owing to their rapid thickening ability, stability, and tolerable taste and smell [1, 3].

Although food thickeners are used for administering oral medications, they have several limitations. For instance, immersion of orally disintegrating (OD) voglibose tablets in food thickeners for 10 min delays disintegration performance and reduces pharmaceutical effects [4]. Additionally, magnesium oxide tablets, which are frequently taken with food thickeners according to a questionnaire survey in care facilities [5], have been detected in the stool of patients who consumed them with food thickeners [6]. Magnesium oxide tablets immersed in food thickeners for 30 min showed an extended disintegration time and reduced laxative action in mice [7]. Additionally, the dosage of magnesium oxide in patients using food thickeners should be higher than that in patients not using food thickeners [8]. Previously, we highlighted the necessity to prevent significantly delayed disintegration or non-disintegration of magnesium oxide tablets by avoiding the immersion of the tablets in food thickeners for a long time; the immersion time should be approximately 1 min [9]. Furthermore, the effects of different types of food thickener components on the disintegration time of tablets are different [9]. Thickened drinks, which represent a feasible alternative, are also commercially available. However, the thickener components of thickened drinks are not disclosed and the effects of thickened drinks on the disintegration of tablets are not clear. In the present study, the effects of thickened drinks on the disintegration time of 40 therapeutic tablets, which are often administered with food thickeners in nursing facilities, was evaluated.

## 2 Materials and methods

### 2.1 Medications and auxiliary foods

In this study, various oral tablets, which are taken with food thickeners according to a questionnaire survey in care facilities [5], were used in this study (Table 1). Specifically, 15 naked tablets, 11 film-coated tablets, 11 OD tablets, 2 enteric tablets, and 1 sugar-coated tablet were used. The powder-type food thickener used for comparison was Tsururinko Quickly (3.0 g/pack; Clinico Co., Tokyo, Japan). Oi Ocha Roasted Green Tea (ITO EN, Tokyo, Japan) was used to dissolve food thickeners. Five different flavors of Ever Smile thickened drinks were used (roasted green tea, green tea, sports drink, black coffee, and apple; Daiwa Can Company, Tokyo, Japan). The contents of the food thickener and thickened drinks are presented in Table 2.

**Table 1:** Medications.

Generic Name	Product Name	Company	Lot Number	Characteristic
Magnesium oxide	Magmitt® Tab. 330 mg	Kyowa Chemical Industry Co., Ltd.	19B028	naked tablet
	Magnesium Oxide Tablet 330 mg “Yoshida”	Yoshida Pharmaceutical Co., Ltd.	B775	naked tablet
	Magnesium Oxide Tablet 330 mg “MOCHIDA”	Mochida Pharmaceutical Co., Ltd.	KE02	naked tablet
	Magnesium Oxide Tablet 330 mg “KENEI”	Kenei Pharmaceutical Co., Ltd.	919709	naked tablet
	Magnesium Oxide Tablet 330 mg “Mylan”	Mylan Inc.	M238AB7	naked tablet
Furosemide	Lasix® Tablet 40mg	Sanofi K.K.	9K190A	naked tablet
	Furosemide Tab. 40mg “TAKEDA TEVA”	Teva Takeda Pharma Ltd.	19L191	film coated tablet
	Furosemide Tablet 40mg“NP”	Nipro Corporation	L631L70	naked tablet
	Furosemide Tablet 40mg “JG”	Nihon Genetic Co., Ltd.	F00169	naked tablet
Amlodipine	Norvasc OD® Tablet 2.5mg	Pfizer Japan Inc.	DC7610	OD tablets
	Amlodin® OD 2.5mg	Sumitomo Dainippon Pharma Co., Ltd.	3205C	OD tablets
	Amlodipine OD 2.5mg "SAWAI”	Sawai Pharmaceutical Co., Ltd.	619902	OD tablets
	Amlodipine OD 5mg "SAWAI”		620402	
	Amlodipine OD 10mg "SAWAI”		620202	
	Norvasc® Tablet 2.5mg	Pfizer Japan Inc.	CN4524	film coated tablet
	Amlodin® 2.5mg	Sumitomo Dainippon Pharma Co., Ltd.	2002C	film coated tablet
	Amlodipine 2.5mg “SAWAI”	Sawai Pharmaceutical Co., Ltd.	119406	film coated tablet
Lansoprazole	Takepron® OD Tablet 15mg	Teva Takeda Pharma Ltd.	EB141	OD tablets
	Lansoprazole OD 15mg “TOWA”	Towa Pharmaceutical Co., Ltd.	D0408	OD tablets
	Lansoprazole OD 15mg “SAWAI”	Sawai Pharmaceutical Co., Ltd.	519903	OD tablets
Famotidine	Gaster® D Tablet 20mg	LTL Pharma	19038T1	OD tablets
	Famotidine OD 20mg “TOWA”	Towa Pharmaceutical Co., Ltd.	A0720	OD tablets
	Famotidine D 20mg “SAWAI”	Sawai Pharmaceutical Co., Ltd.	419905	OD tablets
	Famotidine 20mg “SAWAI”	Sawai Pharmaceutical Co., Ltd.	119503	naked tablet
Sodium Picosulfate	Laxoberon® Tablet 2.5mg	Teijin Pharma Limited.	4157	film coated tablet
	Sodium Picosulfate tablet 2.5mg “IWAKI”	Iwaki Seiyaku Co., Ltd.	97101	film coated tablet
	Sodium Picosulfate tablet 2.5mg “NICHIIKO”	Nichi-Iko Pharmaceutical Co., Ltd.	E00500	film coated tablet
	Sodium Picosulfate tablet 2.5mg “SAWAI”	Sawai Pharmaceutical Co., Ltd.	119703	naked tablet
Warfarin Potassium	Warfarin tablet 1mg	Eisai Co., Ltd.	98A45K	naked tablet
	Warfarin Potassium tablet 1mg “TOWA”	Towa Pharmaceutical Co., Ltd.	D0030	naked tablet
Sodium Valproate	Depakene® Tablet 200mg	Kyowa Kirin Co., Ltd.	875AHJ	film coated tablet
	Valerin® 200mg	Sumitomo Dainippon Pharma Co., Ltd.	2516C	sugar coated tablet
Sodium Ferrous Citrate	Ferromia® 50mg	Sannova Co., Ltd.	93C78S	film coated tablet
	Sodium Ferrous Citrate tablet 50mg “SAWAI”	Sawai Pharmaceutical Co., Ltd.	719927	film coated tablet
	Sodium Ferrous Citrate tablet 50mg “JG”	Nihon Genetic Co., Ltd.	L7L1L70	film coated tablet
Bifidobacterium	LAC-B Tablet	Kowa Company, Ltd.	PL9H	naked tablet
	Biofermin® Tablet	BIOFERMIN	9B282	naked tablet
Lactomin/Bifidobacterium	Lebenin®-S Tablet	Wakamoto Pharmaceutical Co., Ltd.	9810	naked tablet
Aspirin	Bayaspirin® 100mg	Bayer Yakuhin, Ltd	JPS3573	enteric coated tablet
	Aspirin Enteric-Coated Tablet 100mg “TOWA”	Towa Pharmaceutical Co., Ltd.	B0062	enteric coated tablet

OD tablets: orally disintegrating tablet.

**Table 2:** Nutrient compositions of the thickened drinks (100 g).

		Roasted Green tea	Green Tea	Sports Drink	Black Coffee	Apple
Energy	kcal	6	12	24	12	28
Protein	g	0	0	0	0.1	0
Lipid	g	0	0	0	0	0
Carbohydrate	g	1.8	3.2	6.3	3.3	7.4
Sodium	mg	26.7	27	51.8	33	20.6
Potassium	mg	27.8	28	20.4	72	31.2
Calcium	mg	0	0	1.7	1	0
Phosphorus	mg	1.8	1	0	4	1.5
Iron	mg	0	0	0	0	0
Zinc	mg	0	0	0	0.1	0

Apple-flavored drink is 10% juice.

### 2.2 Food thickener preparation

The method of food thickener preparation has been described previously [9]. Briefly, 1.0 or 3.0 g of Tsururinko Quickly was added to roasted green tea (100 mL) and immediately mixed with a spoon. After 2 min of mixing, the solution was used in the experiments.

### 2.3 Line spread test (LST) and pH evaluation

The LST was performed according to the Japanese Dysphagia Diet 2013 guidelines defined by the Dysphagia Diet Committee of the Japanese Society of Dysphagia Rehabilitation (JDD2013) using a plastic measuring disk (Saraya Co., Osaka Japan) [2]. The pH of food thickeners was measured using LAQUAtwin (HORIBA, Ltd., Kyoto, Japan). The thickened drinks were stored at 4 °C. Before the experiments, the drinks were returned to room temperature. LST and pH were examined thrice independently.

### 2.4 Disintegration test

The tablets were immersed in the food thickener or thickened drinks for 1 min. The disintegration time of non-immersed tablets was used as control. The disintegration test was performed according to the method described in the Japanese Pharmacopoeia (17th Edition). The test solutions used were the first (pH 1.2) and second fluids (pH 6.8). As the food thickeners could not be completely removed from the surface of the tablets, the disintegration time was defined as the time at which the contents of the tablets were released [9]. The experiments were performed for a maximum of 2 h; the tablets that were not disintegrated after 2 h were considered “non-disintegrated.” Each test was performed with nine tablets.

### 2.5 Statistical analysis

The results are shown as mean ± standard deviation. Statistical analyses were performed using a one-way analysis of variance (ANOVA) with Tukey–Kramer post-hoc test. Results with a P-value < 0.05 were considered statistically significant. When the tablets were not disintegrated within 2 h, the disintegration time was defined as 2 h.

## 3 Results

### 3.1 LST and pH of the thickened drinks

The LST and pH of the five thickened drinks (roasted green tea, green tea, sports drink, black coffee, and apple) were examined 48 h after opening the bottles (Table 3). The LST values of the thickened drinks were in the range of 45.1–45.9 mm; the pH of the thickened roasted green tea, green tea, and black coffee drinks was 6.5, 6.6, and 5.9, respectively, immediately after opening the bottles. The pH of the sports drink- and apple-flavored thickened drinks was 3.8 and 3.5, respectively. The LST and pH values were maintained for 48 h.

**Table 3:** LST and pH values of the thickened drinks.

		Roasted green tea	Green tea	Sports drink	Coffee	Apple
LST	0 h	45.1 ± 0.4	45.3 ± 0.6	45.8 ± 0.8	45.7 ± 0.7	45.9 ± 0.9
	24 h	45.1 ± 0.2	45.7 ± 1.1	46.1 ± 0.2	45.8 ± 0.7	45.8 ± 1.0
	48 h	46.2 ± 0.8	45.8 ± 0.6	46.7 ± 1.1	45.9 ± 1.4	46.1 ± 0.5
pH	0 h	6.5 ± 0.0	6.6 ± 0.1	3.8 ± 0.0^a,b^	5.9 ± 0.0^a,b,c^	3.5 ± 0.0^a,b,c,d^
	24 h	6.6 ± 0.0	6.7 ± 0.0∗^,a'^	3.8 ± 0.1^a,b^	5.9 ± 0.0^a,b,c^	3.5 ± 0.0^a,b,c,d^
	48 h	6.7 ± 0.1	6.8 ± 0.1	3.8 ± 0.0^a,b^	6.0 ± 0.1^a,b,c^	3.5 ± 0.0^a,b,c',d^

The time shown is the time after opening.

Statistically significant differences among 0, 24, and 48 h are shown at p < 0.05 (∗) (vs. 0 h) (Tukey–Kramer post-hoc test).

Statistically significant differences among all tastes at the same time point are shown at p < 0.001 (a), p < 0.05 (a^'^) (vs. roasted green tea); p < 0.001 (b) (vs. green tea); p < 0.001 (c), p < 0.01 (c^'^) (vs. sports drink); p < 0.001 (d) (vs. coffee) (Tukey–Kramer post-hoc test).

### 3.2 Tablet disintegration time in the thickened roasted green tea drink and food thickeners

#### 3.2.1 Naked tablets

The disintegration times of the 15 naked tablets immersed in the thickened drinks and food thickeners are shown in Table 4. Food thickeners were used at concentrations of 1% (LST: 45.0) and 3% (LST: 32.7). An evaluation of the disintegration time of the non-immersed tablets revealed that 8 tablets disintegrated within 1 min, 6 disintegrated within 1–10 min, and 1 tablet took more than 10 min to disintegrate. The non-immersed magnesium oxide tablets—Magmitt, Mochida, Kenei, and Yoshida—disintegrated in 6–7 s, whereas when immersed in thickened roasted green tea or 1.0% or 3.0% food thickeners, they required 6–14 s to disintegrate. The disintegration time of the non-immersed “Mylan” magnesium oxide tablet was prolonged from 30 s to 2 min when immersed in the thickened drink. In general, the disintegration time of magnesium oxide tablets was similar in 1% or 3% food thickeners or thickened roasted green tea. The non-immersed “Lasix” furosemide tablets fully disintegrated in 18 s, whereas it took 1.5 and approximately 3–3.5 min when immersed in thickened drinks and food thickeners, respectively. The disintegration time of the non-immersed LAC-B Bifidobacterium and warfarin tablets, that is, 18 and 31 s, was slightly prolonged to 23–36 and 45–59 s when immersed in thickened drinks and food thickeners, respectively. Lebenin-S tablets disintegrated in approximately 10.5 min under all conditions. Other naked tablets, which disintegrated within 1–10 min, showed some changes in the disintegration times when immersed in thickened drinks or food thickeners, but the changes were not marked.

**Table 4:** Disintegration time of naked tablets.

Generic Name	Product Name	non-immersion	Thickened Drink	1% Food Thickener	3% Food Thickener
Magnesium Oxide	Magmitt® Tab.	5.7 ± 1.3	11.9 ± 2.8^a"^	7.7 ± 2.9^b'^	7.3 ± 1.2^b"^
	Magnesium Oxide Tablet “MOCHIDA”	5.6 ± 0.9	12.3 ± 4.8^a"^	6.2 ± 1.5^b"^	9.6 ± 1.7^a^
	Magnesium Oxide Tablet “KENEI”	6.6 ± 1.1	13.4 ± 3.9^a"^	5.7 ± 2.8^b"^	6.3 ± 1.6^b"^
	Magnesium Oxide Tablet “Yoshida”	7.4 ± 0.9	14.1 ± 6.5^a'^	8.9 ± 1.5^b^	14.2 ± 3.6^a',c^
	Magnesium Oxide Tablet “Mylan”	29.7 ± 2.5	124.0 ± 91.3	93.6 ± 97.3	118.1 ± 63.6
Furosemide	Lasix® Tablet	18.3 ± 2.0	84.1 ± 12.0^a"^	206.1 ± 40.4^a",b"^	178.4 ± 35.7^a",b"^
	Furosemide Tablet “NP”	175.8 ± 16.5	153.4 ± 13.2^a^	155.1 ± 14.4^a^	185.0 ± 12.5^b",c"^
	Furosemide Tablet “JG”	118.9 ± 9.6	134.3 ± 18.9	150.2 ± 17.4	241.6 ± 51.4^a",b",c"^
Famotidine	Famotidine “SAWAI”	374.0 ± 6.3	424.0 ± 29.9^a^	412.6 ± 21.1	516.6 ± 65.9^a",b",c"^
Sodium Picosulfate	Sodium Picosulfate tablet “SAWAI”	112.2 ± 11.3	106.7 ± 24.6	104.4 ± 18.3	97.2 ± 17.2
Warfarin Potassium	Warfarin tablet	31.3 ± 1.7	44.6 ± 3.6^a'^	49.2 ± 11.6^a"^	59.4 ± 10.7^a",b'^
	Warfarin Potassium tablet “TOWA”	173.8 ± 14.7	265.1 ± 9.2^a"^	307.2 ± 25.6^a",b"^	333.9 ± 19.3^a",b",c^
Lactomin/Bifidobacterium	Lebenin®-S Tablet	625.3 ± 50.6	621.6 ± 53.1	606.0 ± 29.8	621.6 ± 21.6
Bifidobacterium	LAC-B Tablet	18.3 ± 1.1	25.2 ± 2.8^a'^	22.6 ± 2.2	36.0 ± 5.9^a",b",c"^
	Biofermin® Tablet	213.7 ± 16.5	262.7 ± 19.7^a"^	311.8 ± 32.1^a",b"^	316.2 ± 17.0^a",b"^

First fluid was used as the test fluid for all tablets, and all disintegration times are shown in seconds.

Statistically significant differences are shown at p < 0.05 (a), p < 0.01 (a'), p < 0.001 (a'') (vs. non-immersed); p < 0.05 (b), p < 0.01 (b'), p < 0.001 (b'') (vs. thickened drink); p < 0.05 (c), p < 0.001 (c'') (vs. 1% food thickener) (Tukey–Kramer post-hoc test).

#### 3.2.2 Film-coated tablets

The disintegration times of the 11 film-coated tablets immersed in thickened drinks and food thickeners are shown in Table 5. All non-immersed tablets disintegrated in 1–14 min. When the tablets were immersed in thickened drinks or 1% food thickeners, their disintegration times were shorted or remained unaltered. Ferromia and sodium ferrous citrate 50 mg “SAWAI” tablets showed a delay in disintegration by a few minutes when immersed in 3% food thickeners.

**Table 5:** Disintegration time of film-coated tablets.

Generic Name	Product Name	non-immersion	Thickened Drink	1% Food Thickener	3% Food Thickener
Furosemide	Furosemide Tab. 40mg “TAKEDA TEVA”	84.2 ± 25.1	63.3 ± 9.0^a^	58.2 ± 7.1^a'^	63.4 ± 8.2^a^
Amlodipine	Norvasc® Tablet 2.5mg	99.8 ± 18.6	71.6 ± 2.7^a"^	74.7 ± 3.8^a"^	76.9 ± 7.4^a"^
	Amlodin® 2.5mg	136.6 ± 17.7	77.9 ± 24.6^a"^	100.9 ± 15.7^a',b'^	108.1 ± 15.0^a^
	Amlodipine 2.5mg “SAWAI”	56.0 ± 11.4	20.0 ± 4.9^a"^	22.2 ± 4.9^a"^	25.2 ± 1.9^a"^
Sodium Picosulfate	Laxoberon® Tablet 2.5mg	274.8 ± 21.4	207.1 ± 18.1^a"^	192.7 ± 13.5^a"^	216.0 ± 29.9^a"^
	Sodium Picosulfate tablet 2.5mg “IWAKI”	272.6 ± 26.5	221.9 ± 14.6^a"^	221.1 ± 7.0^a"^	225.7 ± 9.5^a"^
	Sodium Picosulfate tablet 2.5mg “NICHIIKO”	323.8 ± 50.9	316.6 ± 29.1	324.1 ± 25.0	324.8 ± 25.5
Sodium Valproate	Depakene® Tablet 200mg	844.6 ± 107.6	815.6 ± 179.2	653.6 ± 227.9	634.1 ± 132.3
Sodium Ferrous Citrate	Ferromia® 200mg	718.8 ± 92.9	683.2 ± 83.3	631.4 ± 98.3	901.7 ± 134.6^a',b",c"^
	Sodium Ferrous Citrate tablet 50mg “SAWAI”	620.3 ± 35.9	635.2 ± 35.3	633.1 ± 40.2	729.7 ± 83.3^a",b',c'^
	Sodium Ferrous Citrate tablet 50mg “JG”	725.1 ± 31.7	729.2 ± 29.7	708.1 ± 47.4	751.1 ± 50.5

First fluid was used as the test fluid for all tablets, and all disintegration times are shown in seconds.

Statistically significant differences are shown at p < 0.05 (a), p < 0.01 (a'), p < 0.001 (a'') (vs. non-immersed); p < 0.01 (b'), p < 0.001 (b'') (vs. thickened drink); p < 0.01 (c'), p < 0.001 (c'') (vs. 1% food thickener) (Tukey–Kramer post-hoc test).

#### 3.2.3 Orally disintegrating tablets

The disintegration times of OD tablets are shown in Table 6. All tablets disintegrated within 40 s, and they showed delayed disintegration when immersed in thickened roasted green tea and food thickeners. In particular, the disintegration of amlodipine OD “SAWAI” tablets was delayed by approximately 5.5 and 12.5 min when immersed in thickened roasted green tea and 3% food thickeners, respectively. The other tablets fully disintegrated in 30 s to 3 min when immersed in thickened drinks and food thickeners.

**Table 6:** Disintegration time of OD tablets.

Generic Name	Product Name	non-immersion	Thickened Drink	1% Food Thickener	3% Food Thickener
Amlodipine	Norvasc OD® Tablet 2.5mg	25.2 ± 1.6	118.3 ± 16.0^a"^	139.8 ± 12.0^a",b'^	148.9 ± 14.9^a",b"^
	Amlodin® OD 2.5mg	22.8 ± 2.2	51.7 ± 9.2^a"^	85.7 ± 14.6^a",b"^	93.1 ± 17.7^a",b"^
	Amlodipine OD 2.5mg “SAWAI”	27.7 ± 3.5	323.8 ± 122.0^a"^	180.8 ± 87.9^a',b'^	747.7 ± 36.9^a",b",c"^
Lansoprazole	Takepron® OD Tablet 15mg	17.9 ± 1.3	34.1 ± 4.0^a"^	30.7 ± 6.6^a"^	45.9 ± 9.2^a",b',c"^
	Lansoprazole OD 15mg “TOWA”	18.8 ± 1.6	39.3 ± 3.4^a"^	35.3 ± 4.6^a"^	53.0 ± 12.3^a",b",c"^
	Lansoprazole OD 15mg “SAWAI”	16.6 ± 1.3	50.9 ± 21.2^a"^	23.6 ± 1.5^b"^	39.7 ± 9.1^a",c^
Famotidine	Gaster® D Tablet 20mg	39.2 ± 3.9	76.2 ± 7.0^a"^	98.8 ± 7.8^a",b"^	122.0 ± 19.2^a",b",c"^
	Famotidine OD 20mg “TOWA”	16.3 ± 2.2	98.7 ± 5.8^a"^	106.7 ± 9.2^a"^	141.2 ± 14.9^a",b",c"^
	Famotidine D 20mg “SAWAI”	20.3 ± 3.6	86.9 ± 9.3^a"^	94.0 ± 19.8^a"^	81.4 ± 14.5^a"^

First fluid was used as the test fluid for all tablets, and all disintegration times are shown is seconds.

Statistically significant differences are shown at p < 0.01 (a'), p < 0.001 (a'') (vs. non-immersed); p < 0.01 (b'), p < 0.001 (b'') (vs. thickened drink); p < 0.05 (c), p < 0.001 (c'') (vs. 1% food thickener) (Tukey–Kramer post-hoc test).

#### 3.2.4 Enteric- and sugar-coated tablets

The disintegration times of the enteric- and sugar-coated tablets are shown in Table 7. Although the two enteric-coated aspirin tablets did not disintegrate in the first fluid (pH 1.2) even after 2 h, they fully disintegrated within 7–10 min in the second fluid (pH 6.8) regardless of the presence of food thickeners. Sugar-coated valerin tablets, which disintegrated in 11.5 min when not immersed in food thickeners, required approximately 10 min to disintegrate in thickened roasted green tea and food thickeners.

**Table 7:** Disintegration time of enteric- and sugar-coated tablets.

Generic Name	Product Name	Test Fluid	non-immersion	Thickened Drink	1% Food Thickener	3% Food Thickener
Aspirin	Bayaspirin® 100mg	First fluid	>120	>120	>120	>120
		Second fluid	9.5 ± 2.3	9.0 ± 1.9	9.3 ± 1.5	9.2 ± 1.4
	Aspirin Enteric-Coated Tablet 100mg “TOWA”	First fluid	>120	>120	>120	>120
		Second fluid	8.7 ± 1.0	7.2 ± 0.7^a'^	8.3 ± 0.8	8.2 ± 1.1
Sodium Valproate	Valerin® 200mg	First fluid	11.5 ± 0.6	9.7 ± 0.5^a"^	10.3 ± 0.2^a"^	10.2 ± 0.7^a"^

All disintegration times are shown in minutes.

Statistically significant differences are shown at p < 0.01 (a'), p < 0.001 (a'') (vs. non-immersed) (Tukey–Kramer post-hoc test).

### 3.3 Effect of different thickened drinks on the disintegration time of tablets

**Figure 1:**
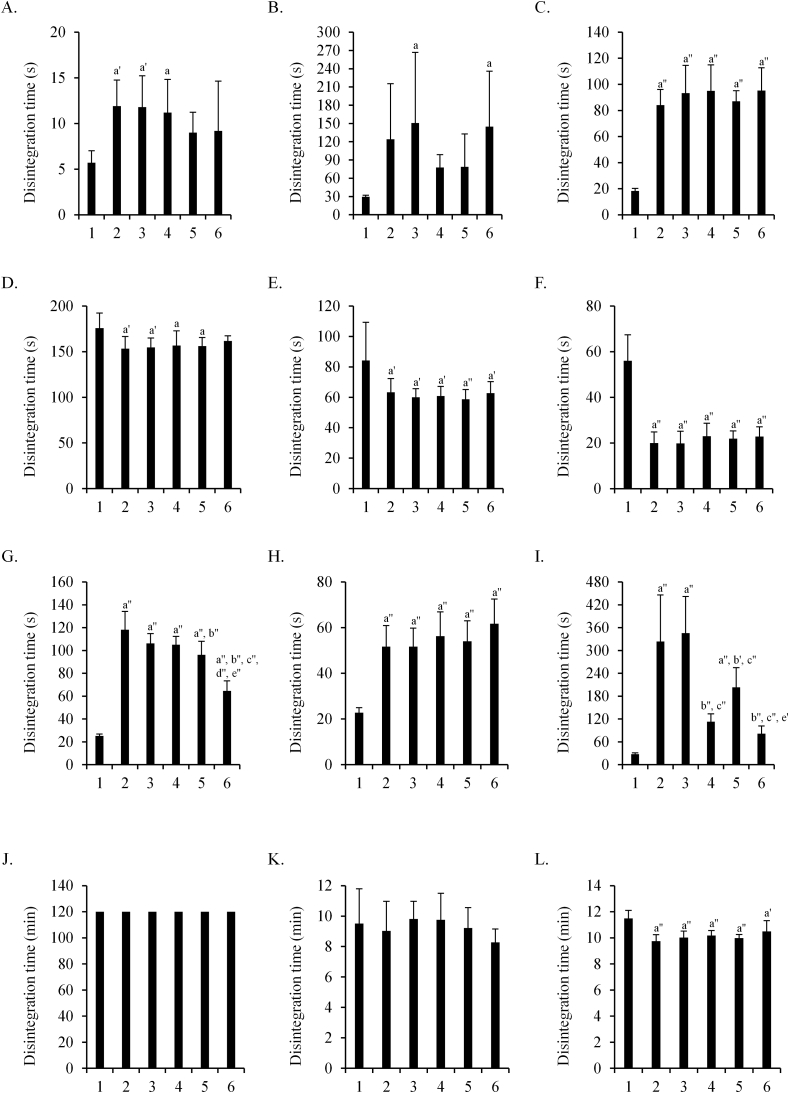
Comparison of the disintegration time among thickened drinks of various flavors. Effects of five thickened drinks on the disintegration time of the tablets were examined. A: Magmitt® tablet 330 mg, B: Magnesium oxide tablet 330 mg “Mylan”, C: Lasix® tablet 40 mg, D: Furosemide tablet 40 mg “NP”, E: Furosemide tablet 40 mg “TAKEDA TEVA”, F: Amlodipine 2.5 mg “SAWAI”, G: Norvasc OD® tablet 2.5 mg, H: Amlodin® OD 2.5 mg, I: Amlodipine OD 2.5 mg “SAWAI”, J and K: Bayaspirin® 100 mg, L: Valerin® 200 mg. 1: non-immersed, 2: roasted green tea, 3: green tea, 4: sports drink, 5: coffee, 6: apple. Test fluids were first fluid (A-J and L) and second fluid (K). The disintegration time of non-immersed tablets and of those immersed in roasted green tea-flavor thickened drink is the same as mentioned in Tables 4, 5, 6, and 7. Statistically significant differences in the disintegration times of non-immersed tablets and of those immersed in the five thickened drinks are shown at p < 0.05 (a), p < 0.01 (a'), p < 0.001 (a'') (vs. non-immersed); p < 0.01 (b'), p < 0.001 (b'') (vs. roasted green tea); p < 0.001 (c'') (vs. green tea); p < 0.001 (d'') (vs. sports drink); p < 0.01 (e'), p < 0.001 (e'') (vs. coffee) (Tukey–Kramer post-hoc test).

The effects of the thickened drinks on the disintegration time of the tablets were examined (Figure 1). Although Norvasc OD tablets showed delayed disintegration in all types of thickened drinks, the effects of the apple-flavored drink were less pronounced. Moreover, the delay in the disintegration of amlodipine OD “SAWAI” tablets immersed in sports-, coffee-, and apple-flavored drinks was shorter than that in the roasted green tea and green tea thickened drinks. The disintegration time of the other tablets was similar among all the thickened drinks.

### 3.4 Comparison of the disintegration time of amlodipine OD “SAWAI” tablets at three doses

**Figure 2:**
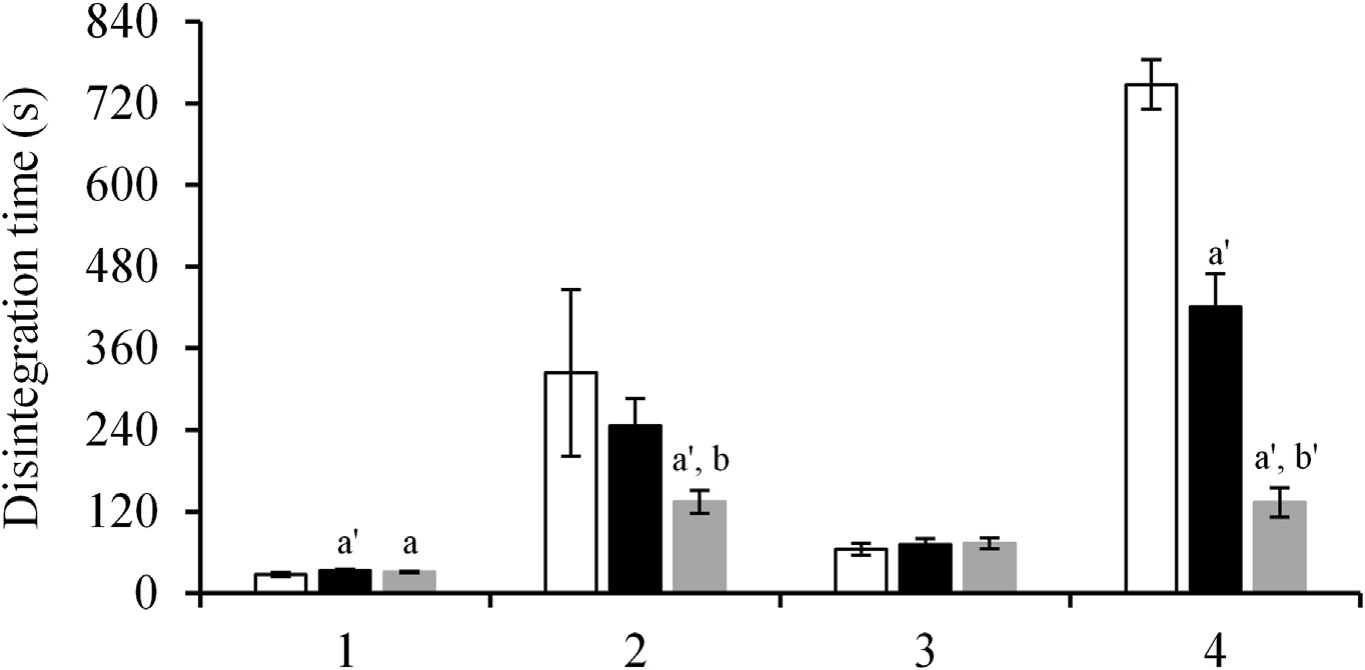
Comparison of the disintegration time of amlodipine OD “SAWAI” various doses. White column: 2.5 mg, black column: 5 mg, gray column: 10 mg of amlodipine OD “SAWAI”. 1: non-immersed, 2: roasted green tea (thickened drink), 3: apple (thickened drink), 4: 3% food thickener. The disintegration time of 2.5 mg tablets is the same as mentioned in Table 6. Statistically significant differences in the disintegration times of 2.5, 5, and 10 mg tablets are shown at p < 0.01 (a), p < 0.001 (a') (vs. 2.5 mg tablets); p < 0.01 (b), p < 0.001 (b') (vs. 5 mg tablets) (Tukey–Kramer post-hoc test).

The disintegration times of amlodipine OD “SAWAI” tablets at three doses (2.5, 5, and 10 mg) were compared when these tablets were immersed in thickened drinks (roasted green tea and apple) and 3% food thickener. The disintegration times of amlodipine OD “SAWAI” tablets of different doses immersed in apple-flavored thickened drink were similar. However, the disintegration time of tablets immersed in roasted green tea-flavored thickened drink and 3% food thickener increased in the following order: 10 mg < 5 mg < 2.5 mg (Figure 2).

## 4 Discussion

Powder-type food thickeners can be used in a personalized manner, per the needs of the patient; however, the thickeners should be prepared every time they are used. Therefore, thickened drinks represent a feasible and ready-to-use alternative, if their thickening strength is appropriate for patients with dysphagia.

Thickening strength can be defined using LST values (mm) according to the Japanese Dysphagia Diet 2013 guidelines as extremely thick (LST value: 30–32), moderately thick (LST value: 32–36), and mildly thick (LST value: 36–43) [2]. Herein, the LST of the thickened drinks tested was approximately 45–46. Taking into consideration that moderately thick drinks were first tried in patients with dysphagia after a stroke [2], thickened drinks may be appropriate for patients with mild dysphagia. Here, although the thickened drinks maintained their stability when stored at 4 °C, with unchanged line spread and pH values for 48 h after opening the bottles, the potential risk of bacterial contamination with prolonged storage should be considered.

In this study, the effect of thickened drinks on the disintegration times of 40 tablets, which are taken with food thickeners according to a questionnaire survey in care facilities [5], was examined. The roasted green tea-flavor drink was used as the main test thickened drink to avoid potential confounder elements, such as caffeine and various ions in the other drinks. Several tablets showed a significant delay in disintegration when immersed in the thickened drinks; however, the tablets whose disintegration time was short in the absence of thickened drinks or food thickeners tended to show delayed disintegration. Additionally, the effects of the thickened drinks and food thickeners were similar. In particular, the disintegration time of all OD tablets was longer (maximum 12.5 min) than that of the non-immersed tablets when immersed in thickened drinks and food thickeners. The retention time of medications in the stomach ranges from 5 min to 2 h [10]; therefore, the effects of food thickeners on OD tablets might be low. However, OD tablets generally rapidly disintegrate in the oral cavity by saliva, and they could be taken without water. When food thickeners were used for taking OD tablets, it is difficult to gain the advantage. It might be safe for patients taking OD tablets to take them without food thickeners or other types of tablets with food thickeners.

Masuda classified OD tablets on technical bases such as manufacturing methods and additives used [11]. The characteristics of such OD tablets were different. This might also be the reason for the difference in the rate of disintegration delay among OD tablets. The disintegration time of amlodipine OD “SAWAI” at different doses increased in the following order: 10 mg < 5 mg < 2.5 mg. The same additives are used in these tablets, but their weight and size are different. The diameter, thickness, and weight in the 2.5 mg tablets were 6.0 mm, 2.9 mm, and 85 mg; 5 mg tablets were 7.0 mm, 3.0 mm, and 120 mg; and 10 mg tablets were 8.5 mm, 4.1 mm, and 240 mg, respectively. A longer time is required for food thickeners to penetrate larger OD tablets; therefore, the disintegration of 10 mg tablets immersed in food thickeners could be faster than that of the other dose tablets. Furthermore, the sports drink- (pH 3.8) and apple-flavored thickened drinks (pH 3.5), with weak acidic pH, had less effect on the disintegration time of tablets than roasted green tea- (pH 6.5) and green tea-flavored thickened drinks (pH 6.6). The pH of thickened drinks might affect the disintegration time of tablets. Further studies are warranted to better understand the underlying cause of these differences.

We previously demonstrated that the disintegration pattern of several types of magnesium oxide tablets was different between xanthan gum- and guar gum-based food thickeners [9], an effect that could be dependent on the components of the food thickener. Although the company that markets the thickened drinks does not disclose the thickening components used, the observed effects of the thickened drinks on the disintegration time of tablets were similar to those of xanthan gum-based food thickeners. However, to decide whether thickened drinks can be used to take medications, knowledge of the components of thickened drinks is crucial. Additionally, the disintegration times of most tablets were not significantly different among the five thickened drinks, which provide a broad spectrum of flavor possibilities for patients to select according to their preferences. However, it is noteworthy that the pharmaceutical effects of medications change with the components of drinks.

In conclusion, the thickened drinks evaluated in this study may be useful for patients with mild dysphagia to help them take medications, representing a potential ready-to-use alternative to food thickeners. Our study provides valuable information for pharmacists and clinicians to decide the most suitable way to deliver medications to patients with dysphagia.

## Declarations

### Author contribution statement

Taisuke Matsuo-Conceived and designed the experiments; Performed the experiments; Analyzed and interpreted the data; Contributed reagents, materials, analysis tools or data; Wrote the paper.

Takashi Tomita-Conceived and designed the experiments; Contributed reagents, materials, analysis tools or data.

Chinatsu Sato-Performed the experiments; Analyzed and all authors interpreted the data.

Yasuyuki Sadzuka-Contributed reagents, materials, analysis tools or data.

### Funding statement

This work was supported by the grants-in-aid for scientific research program (KAKENHI) of the 10.13039/501100001691Japan Society for the Promotion of Science (grant number JP19K11283).

### Data availability statement

Data included in article/supplementary material/referenced in article.

### Declaration of interests statement

The authors declare no conflict of interest.

### Additional information

No additional information is available for this paper.
